# The Training of Memory

**Published:** 1915-10-16

**Authors:** 


### The Hospital

The Workers' Newspaper of Administrative Medicine and Institutional Life, Administration, National Insurance and Health.

No. 1531, Vol. LIX. SATURDAY, OCTOBER 16, 1915. PRICE ONE PENNY.

### THE TRAINING OF MEMORY.

If we may judge from the advertisements, there must be few defects or weaknesses of the human mind or body for which either Nature or art has failed to provide a remedy. For the ills of the flesh there have always been many healers, and of recent years the less tangible misfortunes of the spirit seem to have stimulated enterprise. The evidence of this usually appears in confident announcements decorated with the stern features of a gentleman who professes to be a mind-builder and who undertakes to furnish his clients with the qualities which will ensure success in life. We should be very reluctant to deny that these systems often contain good advice, though we fancy that equal value can be obtained from more homely sources and on less expensive terms. As for the Napoleonic results which are promised, it may be permissible to hint a doubt, and, in any event, the world just now is hardly anxious for the multiplication of the superman.

On a less ambitious plane some discussion has recently taken place relative to the cultivation of the faculty of memory, and there are so many who deplore their feebleness in this direction that the subject is worthy of some attention. Indeed it is somewhat astonishing to know that in teaching institutions, where so much depends on successful use of the memory, the function rarely receives anything like ordered study. Yet it must be subject to laws and must be capable of yielding improved results in so far as the conditions prescribed by these laws are satisfied. To say this is not to suggest that there are not in this direction, as in others, degrees of natural capacity. Experience shows very plainly that personal endowment, here as elsewhere, is a large factor, and that to proclaim all men to be equal in the value of their memory possibilities is mere speculative talk divorced from fact. Whether this difference depends on properties of cerebral tissue which can be defined and estimated is perhaps doubtful, and the question is hardly one of practical moment. But even if an anatomical basis is accepted, it in no measure challenges the proposition that the memory function, even as others, is conditioned by certain rules, and that by attention to these rules the function may be cultivated and improved.

As a somewhat rough definition memory may perhaps be described as the art of reviving at will impressions made on the brain by such stimuli as words, events, and ideas. Manifestly the first element of success in the process is that the impression shall be made firmly and clearly. And it is here, we take it, that to a very large extent the difference between different individuals resides. Whether by a special quality of brain tissue or as a result of some other factor there are those—a much envied few—in whom automatically and without conscious effort impressions even from isolated and passing stimuli are indelibly cut and permanently sealed. Such fortunate persons have, as it were, a photographic memory. Obviously for those not so gifted—the enormous majority—there is the plain indication that effort must be made to deepen the impressions to which some relative imperfection of brain tissue forbids a ready and permanent reception. It is a commonplace to say that this means the application of attention to the impressions we desire to retain. "Pay attention" is the exhortation which falls upon every juvenile ear, and every serious student admits its necessity. But much less frequent is it to hear of the need for the revival and repetition of impressions. Yet in exercises of this order are to be secured the most certain methods of fixing a habit even upon nervous structures which are not naturally receptive or tenacious. No doubt some attention, in the shape of revision, is paid to this need in the days of scholastic discipline. But something more than this is needed for him who would make his memory a successful mental instrument, namely—the revival of the original impression, not by repetition of the stimulus from without, but by conscious effort from within. In other words, by an effort of the will, repeated again and again, there must be fixed securely a result which but for this process would prove a mere temporary and passing picture. Those who would fain remember must have not the mere *wish*, but the *will* to remember, and the will must be exercised in proportion to the weakness of the natural capacity. This is probably a hard saying for those who act on the assumption that a good memory is a gift of the gods, and that the absence of it is beyond redress.

There is an art also in the practice of repetition, and this especially for the student who is called upon to remember facts and words and events in series. It is that the impressions which these produce shall be revived always in one and the same order. This truth is always before us, yet many miss it. Most people manage to remember the English alphabet, and it is really a considerable memory achievement. Twenty-six separate signs and sounds no one of which suggests its fellows! How is the task accomplished? It is accomplished by repetition, and by repetition always in the same order. Thus is cultivated an ordered habit of the brain centres, and it is sufficient to commence the movement to ensure completion of the series. If anyone doubts this proposition let him try to repeat the alphabet not from A to Z, but from Z to A. In such a process all the memories indeed are there, but their revival is a slow and halting performance because it is an attempt to reverse the order in which the brain habit has been established. There are other conditions which govern memory culture and to these we may perhaps return, but none is so important as the two here discussed. We commend the consideration of them to those who have hitherto made little effort to redress a weakness which they ostentatiously deplore.

